# Risk factors associated with osteonecrosis of femoral head after internal fixation of femoral neck fracture:a systematic review and meta-analysis

**DOI:** 10.1186/s12891-019-2990-5

**Published:** 2019-12-29

**Authors:** Jing-Li Xu, Zheng-Rong Liang, Bing-Lang Xiong, Qi-Zhao Zou, Tian-Ye Lin, Peng Yang, Da Chen, Qing-Wen Zhang

**Affiliations:** 1 The First Clinical College, Guangzhou University of Chinese Medicine, Guangzhou, China; 2 The First Clinical Medicine School of Jinan University, Guangzhou, China; 3 The Sun Yat-sen University Cancer Center, Guangzhou, China; 4 The First Affiliated Hospital of Guangzhou University of Chinese Medicine, No.16, Ji Chang Road, Baiyun District, Guangzhou, 510405 China

**Keywords:** Femoral neck fracture, Internal fixation, Osteonecrosis of femoral head, Risk factors, Systematic review

## Abstract

**Background:**

Although the risk factors associated with osteonecrosis of femoral head (ONFH) after internal fixation of femoral neck fracture (IFFNF) have been frequently reported, the results remain controversial. Therefore, its related risk factors were systematically evaluated and meta-classified in this study.

**Methods:**

Literature on risk factors of ONFH caused by IFFNF was retrieved in PubMed, Embase and Cochrane Library due June 2019. Review Manager 5.3 software was applied to data synthesis, and Stata 13.0 software was adopted for analyses of publication bias and sensitivity.

**Results:**

A total of 17 case-control studies with 2065 patients were included. The risk of ONFH after IF was 0.40-fold higher in patients with Garden III-IV FNF than that in patients with Garden I-II (OR: 0.40, 95%CI: 0.29–0.55). The risk of OFNH with retained IF was uplifted by 0.04 times (OR: 0.04, 95%CI: 0.02–0.07). There was nonsignificant relationship between gender and ONFH after IFFNF (OR: 1.27, 95%CI: 0.84–1.94). Moreover, ONFH after IFFNF presented no association with age (OR:1.66, 95%CI: 0.89–3.11), injury-operation interval (OR:1.29, 95%CI: 0.82–2.04), fracture reduction mode (OR:1.98, 95%CI: 0.92–4.26), preoperative traction (OR:1.69, 95%CI: 0.29–9.98) and mechanism of injury (OR:0.53, 95%CI: 0.06–4.83). Egger’s and Begg’s tests indicated a publication bias (*P* = 0.001).

**Conclusion:**

It was demonstrated that Garden classification and retained IF were important influencing factors of ONFH after IFFNF. Gender, age, injury-operation interval, fracture reduction mode, preoperative traction and the mechanism of ONFH were irrelevant to the complication.

## Introduction

FNF is a serious traumatic disease, accounting for about 50% of hip fractures. It mainly occurs after falls in the elderly, and is more common in middle-aged people due to violent injuries [1]. In the United States, the incidence of FNF is 63.3 per million in women and 277 per million in men [2]. Although detailed epidemiological data yet have not been available in China, it is estimated that a significant number of this group suffer from FNF. With the aging population increasing and the social modernization forging ahead, the incidence of FNF is on the rise in this country.

Though various treatments for FNF have sprung up, IF with autologous hip joint is still the mainstream [3–5]. ONFH, or avascular necrosis of femoral head, is the ultimate consequence of impaired blood supply. ONFH and nonunion are two major complications after IFFNF, especially in the young group [6, 7]. As the technology of IF advances, the healing rate of FNF has remarkably improved [8–10]. However, recent years have witnessed a nonsignificant drop of the occurrence of ONFH after IFFNF [6, 11].

The risk factors of ONFH after IFFNF have been reported by many lately, but the results of studies are divergent and even controversial. In this study, we retrieved the published literature on ONFH after IFFNF, extracted high-relevant data for a systematic review and meta-analysis, and evaluated the significance of the filtered risk factors, in a bid to provide a reference for the prevention and treatment of this complication.

## Methods

### Search strategies

This study was executed in line with the guidelines of the Preferred Reporting Items for Systematic Reviews and Meta-Analyses (PRISMA) [12] and reported based on the guidelines developed by the Meta-Analysis of Observational Studies in Epidemiology group [13]. Because all the analyses were performed on the basis of previous published studies, no ethical approval or patient consent was required. In the initial screening, 2 investigators (J-LX and B-LX) conducted the main search in the electronic databases of PubMed, Embase and Cochrane Library to retrieve eligible articles on ONFH-related risk factors after IFFNF from the inception of the databases to June 2019, without restrictions to languages, publication types or regions. The combined terms of Medical Subject Headings (MeSH) and non-MeSH were searched as follows: femoral neck fractures, femoral neck fracture, femur neck fractures, femur neck fracture, femur head necrosis, femur head necroses, head necrosis femur, necrosis femur head, aseptic necrosis of femur head, necrosis aseptic of femur head, necrosis avascular of femur head, ischemic necrosis of femoral head, avascular necrosis of femoral head, primary avascular necrosis of femur head,fracture fixation, internal, and internal fixation. A third investigator irrelevant to the initial procedure was consulted in case of any discrepancy.Taking the pubmed database as an example, the literature search strategy is shown in Table 1.

**Table 1:** The Pubmed database literature search strategy

#1	“Femoral Neck Fractures”[Mesh]
#2	Femoral Neck Fractures
#3	Femoral Neck Fracture
#4	Femur Neck Fractures
#5	Femur Neck Fracture
#6	#1 OR #2 OR #3 OR #4 OR #5
#7	“Femur Head Necrosis”[Mesh]
#8	Femur Head Necrosis
#9	Femur Head Necroses
#10	Head Necrosis, Femur
#11	Necrosis, Femur Head
#12	Aseptic Necrosis of Femur Head
#13	Necrosis, Aseptic, of Femur Head
#14	Necrosis, Avascular, of Femur Head
#15	Ischemic Necrosis Of Femoral Head
#16	Femoral Head, Avascular Necrosis Of
#17	Avascular Necrosis Of Femoral Head, Primary
#18	Avascular Necrosis of Femur Head
#19	#7 OR #8 OR #9 OR #10 OR #11 OR #12 OR #13 OR #14 OR #15 OR #16 OR #17 OR #18
#20	“Fracture Fixation”[Mesh]
#21	Fracture Fixation
#22	Fixation
#23	Internal fixation
#24	#20 OR #21 OR #22 OR #23
#25	#6 AND #19 AND #24

### Study selection criteria

Two independent investigators (J-LX and Z-RL) analyzed the initially selected articles to verify their relevance with the topic of risk factors and ONFH after IFFNF. Studies had to fulfill the following criteria for inclusion: outcome was femoral head necrosis;internal fixation for femoral neck fracture;study design included case-control, retrospective,and prospective cohorts, and cross-sectional studies;participants were selected without limitations to regions, ages or social status.Trials were excluded according to following identifications: duplicate or overlapping data, animal experiments, conference abstracts, letters and review articles. In case of any disagreement the results were discussed and unified by senior authors.

### Data extraction

Data from the included studies were extracted and independently categorized by 2 of the authors (J-LX and Q-ZZ) in a predefined data extraction form. All disagreements were resolved by discussion. Design information, baseline population characteristics (mean age, sample size and country), surgical approach, risk factors from all included studies were stratified into a standardized evidence table. All the data were rechecked to ensure accuracy. Study selections were shown in a PRISMA flow diagram.

### Methodological quality assessment

The methodological quality of the included studies was evaluated by 2 independent reviewers (J-LX and T-YL) based on the items of modified Newcastle-Ottawa Scale (NOS) [14], comprising patient selection, study group comparability and outcome assessment. The observational studies scored 0 to 9. Divaricate opinions were discussed among the authors.

### Statistical analysis

The meta-analysis and statistical analysis were performed using Cochrane Collaboration Review Manager software (RevMan version 5.3, Nordic Cochrane center, Copenhagen, Denmark). The odds ratio (OR) was utilized as a statistical analysis. The *I*-square (*I*^2^) test was adopted to evaluate the influence of heterogeneity on the output of meta-analysis. *I*^2^ values of 0, 25, 50 and 75% represented no, low, medium and high heterogeneity, respectively. According to the Cochrane review guidelines, severe heterogeneity of *I*^2^ ≥ 50% required the utilization of random-effect models. Otherwise, the fixed effect model was approved. *P* value less than 0.05 was accepted as statistical significance. Sensitivity analysis [15] was conducted by study removal approach to evaluate the quality and consistency of the results. Funnel plots were visually checked, and Egger and Begg linear regression tests of publication bias were carried out by Stata 13.0 software.

## Results

### Study selection process

**Fig. 1:**
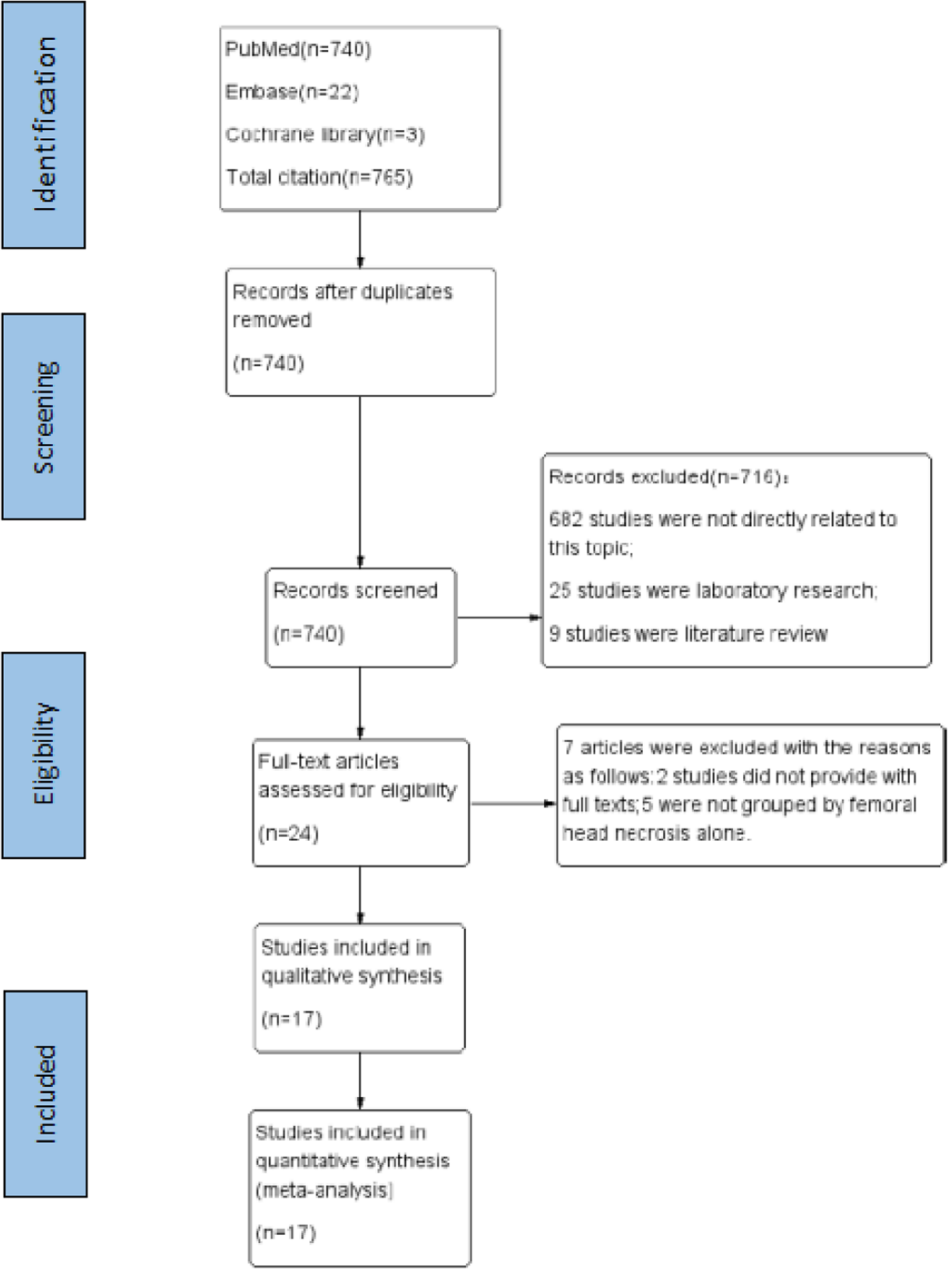
The flow diagram of literature selection

As a result, 765 references were initially retrieved, 740 were left after eliminating duplicate literature; and then 716 without high-relevant to our topic were discarded by reading titles and abstracts, and 24 studies remained. Finally, 7 full-text articles were abandoned because of the following reasons: 2 studies did not provide with full texts; 5 studies lacked the independent groups of ONFH. Therefore, 17 observational studies with 2065 patients were included in the meta-analysis. The flow chart describing the selection process of the study was shown in Fig. 1.

### Study characteristics and methodological quality

The 17 included references were case-control studies, with the publication years differing from 2005 to 2019. Nine were conducted in China (including 1 in Taiwan), 2 in South Korea, and 1 in Malaysia, Chile, France, Saudi Arabia, UK and Brazil, respectively. In the selected clinical trials, the sample sizes varied between 29 and 277 participants.12 studies reported cannulated screw internal fixation, 3 studies reported cannulated screw fixation or dynamic hip screw fixation, 1 study reported dynamic hip screw fixation, and 1 study reported cannulated screw fixation or cannulated screw combined with dynamic hip screw fixation or locking compression plate fixation.2 studies reported preoperative traction,1 study reported skeletal traction, and another study did not indicate traction type;3 studies reported the status of implants,including removal and retention of implants in the body.2 studies reported the mechanism of injury,including high-energy injuries and low energy injuries;3 studies reported fracture reduction mode,including open reduction and closed reduction.The basic characteristics of the 12 of them were shown in Table 2. In addition, all the studies were evaluated as high methodological quality in accordance with the NOS.

**Table 2:** Characteristics of the Included Studies

Included studies	Study design	Country	Studycharacteristics	Follow up (months)	Factors	NOS score
Wang T 2014 [16]	Case–control	China	61 males, 18–68 y	6–90	Age, sex,Garden classification, reduction quality,reduction methods, injury-operationinterval, preoperative traction, weight-bearingtime, implant removal	9
Khoo CCH 2014 [17]	Case–control	Malaysia	39 males, 30–59 y	Unclear	Age, injury-operationinterval, fracture location, integrity of posterior cortex, adequacy of fracture reduction	6
Kang JS 2016 [18]	Case–control	Korea	Unclear, 16–18 y	24–148	Age, injury-operationinterval, osteoporosis, displacement, quality of reduction, firm fixation	6
Wang C 2015 [19]	Case–control	China	62 males, 51.9 ± 9.9 y	34–41	Age, gender, length of stay, fracture laterality, mechanismof injury, procedure delay, duration of surgery, implantconfiguration, interval to full weight-bearing, preoperativeGarden classification, preoperative traction, postoperativevisual analog scale (VAS), Parker score, implant status,residual displacement	8
Zhang C 2017 [20]	Case–control	China	Unclear, 50–70 y	36–48	Age, sex, ASA scale, laterality (L/R), body mass index (BMI), BMD, Garden classification, Bone mineral density, total cholesterol (TC), triglyceride (TG), high density lipoprotein (HDL), low density lipoprotein (LDL), apolipoprotein A1 (Apo-A1), apolipoprotein B (Apo-B)	6
Schweitzer D 2013 [21]	Case–control	Chile	22 males, 46.45 ± 11.59y	24–144	Age, injury-operation interval, anatomicreduction, mechanism of injury, fracture reduction mode	9
Simona P 2008 [22]	Case–control	France	Unclear	Unclear	Garden classification, Pauwels classification	6
Ai ZS 2013 [23]	Case–control	China	44 males, > 45 y	28–60	Age, gender, type of fracture(Garden classification), timing of the reduction, quality ofthe reduction (Garden classification), postoperative durationto full weight bearing, implant status (removal vs. maintenance),preoperative traction, fracture side	8
Wang CT 2018 [24]	Case–control	Taiwan, China	43 males, 50–60 y	12–96	Garden classification, reduction quality,Pauwels classification	6
Zeng XS 2017 [25]	Case–control	China	142 males, 50–94 y	37–46	Age, sex, ASA scale, laterality (L/R), BMI, FNBMD, Garden classification, Garden index, injury-operation interval, weight-bearing activity time, TC, TG, HDL, LDL, Apo-A1, Apo-B	9
Koaban S 2019 [26]	Case–control	Saudi Arabia	60 males, 18–70 y	> 13	Gender, presence of comorbid conditions, mode of injury, multiple trauma, Garden classification,fracture side, time of fixation, type of reduction, full weight-bearing follow-up	9
Razik F 2012 [27]	Case–control	UK	Unclear	Unclear	Fracture side, time of fixation, fracture classification, mechanism, complications, method of fixation	7
Schwartsmann CR 2014 [28]	Case–control	Brazil	56 males, 18–70 y	64.6	Sex, age, Garden grade,time of surgery, reduction	7
Zhang YL 2016 [29]	Case–control	China		21.6 ± 6.0	Garden types	6
Jo S 2016 [30]	Case–control	Korea	25 males, 16–69 y	24–75	Anatomical classification, Garden classification, Pauwels classification	6
Zhao HX 2016 [31]	Case–control	China	61 males, > 18 y	21.6 ± 6.0	Sex, age, Garden grade, time of surgery, Garden index	7
Mao YJ 2005 [32]	Case–control	China	134 males, 13–70 y	12–101	Sex, age, Garden classification, reduction quality, injury-operationinterval, preoperative traction, weight-bearingtime, implant removal, fracture reduction mode	9

*ASA* American Society of Anesthesiologists; *BMD* Bone mineral density; *TC* Total Cholesterol; *TG* Triglyceride; HDL High Density Lipoprotein; *LDL* Low Density Lipoprotein; *Apo-A1* Apolipoprotein A1; *Apo-B* Apolipoprotein B; *BMI* body mass index; *FNBMD* femoral neck bone mineral density

### Risk factors of ONFH after IFFNF

#### Gender

**Fig. 2:**
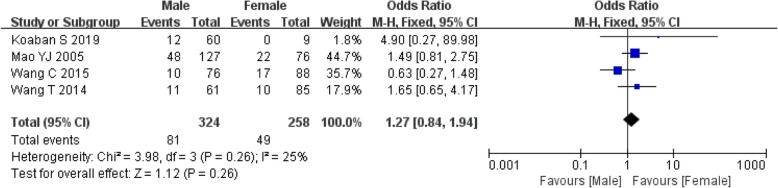
Meta-analysis results of the correlation between gender and ONFH after IFFNF.

Of the 16 included studies, 4 reported [16, 19, 26, 33] the correlation between gender and ONFH after IFFNF. Because of nonsignificant heterogeneity among the studies, the fixed effect model was utilized (*I*^2^ = 25%, *P* = 0.26). However, nonsignificant differences in gender were unveiled between ONFH patients and normal ones (OR: 1.27, 95%CI: 0.84–1.94, *P* = 0.26). (Fig. 2).

#### Age

**Fig. 3:**
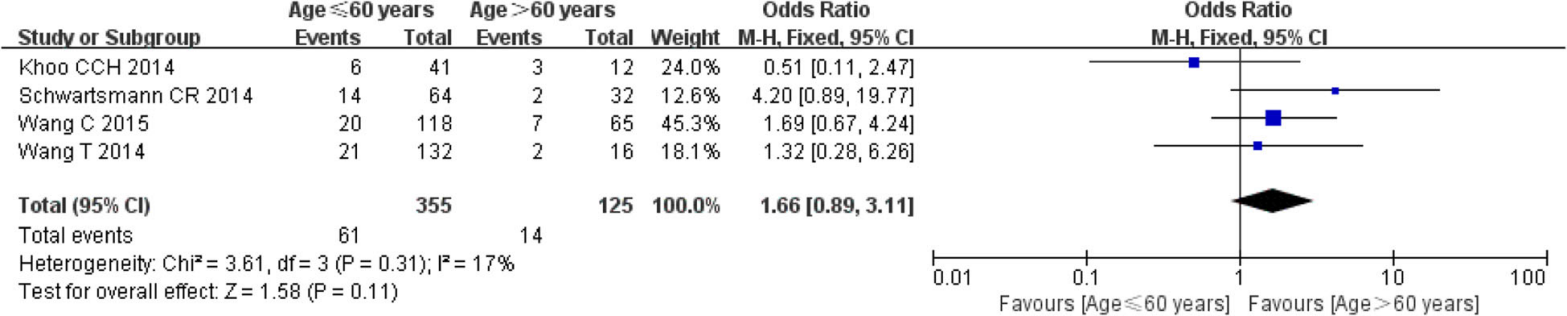
Meta-analysis results of the correlation between age and ONFH after IFFNF.

Data on age were available for the meta-analysis from 4 studies [16, 17, 19, 28], and nonsignificant heterogeneity was presented among the studies (*I*^2^ = 17%, *P* = 0.31). Therefore, the fixed effect model was applied. However, differences in age between ONFH patients and normal ones were still nonsignificant (OR: 1.66, 95%CI: 0.89–3.11, *P* = 0.11). (Fig. 3).

#### Garden classification

**Fig. 4:**
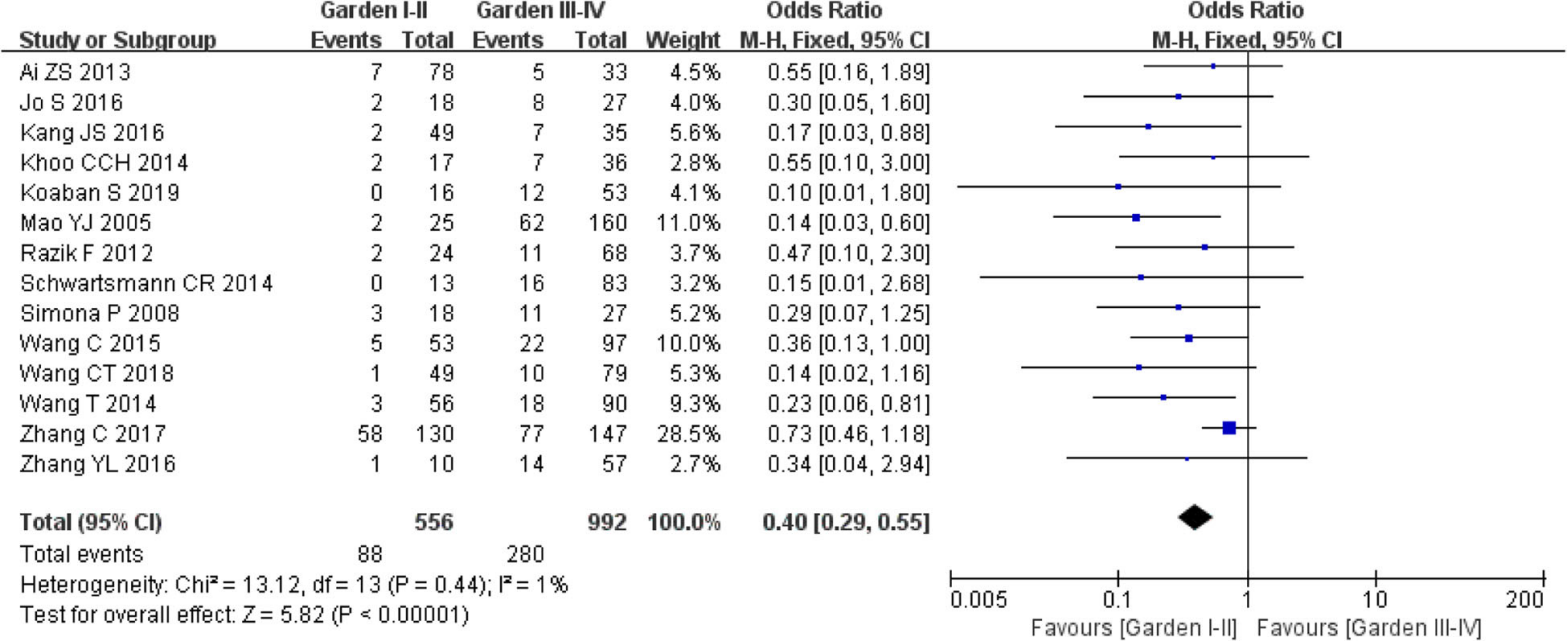
Meta-analysis results of the correlation between Garden classification and ONFH after IFFNF.

Data on Garden classification were available for the systematic analysis from 14 studies [16–20, 22–24, 26–30, 32]. The fixed effect model was chosen due to nonsignificant heterogeneity in intra-study comparisons (*I*^2^ = 1%, *P* = 0.44). The outcome manifested a statistically significant difference in the item between the two patient groups (OR: 0.40, 95%CI: 0.29–0.55, *P* < 0.00001). (Fig. 4).

#### Injury-operation interval

**Fig. 5:**
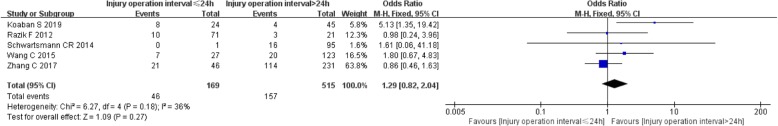
Meta-analysis results of the correlation between injury-operation interval and ONFH after IFFNF.

Five references [19, 20, 26–28] concerning the data on injury-operation interval were available for the analysis^.^ The fixed effect model was used as nonsignificant heterogeneity was found in intra-study comparisons (*I*^2^ = 36%, *P* = 0.18). No statistically significant difference in this index was shown between the two patient groups (OR: 1.29, 95%CI: 0.82–2.04, *P* = 0.27). (Fig. 5).

#### Fracture reduction mode

**Fig. 6:**
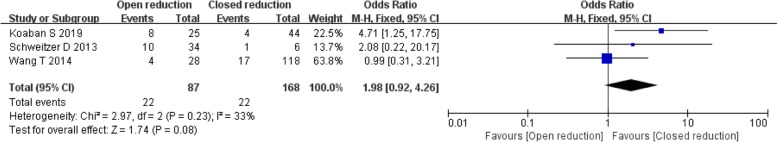
Meta-analysis results of the correlation between fracture reduction mode and ONFH after IFFNF.

Three studies [16, 21, 26] containing statistics on fracture reduction mode were available for the analysis using the fixed effect model, without significant heterogeneity among the studies (*I*^2^ = 33%, *P* = 0.23). The results exhibited no statistically significant differences in fracture reduction mode between the two patient groups (OR:1.98, 95%CI: 0.92–4.26, *P* = 0.08). (Fig. 6).

#### Preoperative traction

**Fig. 7:**
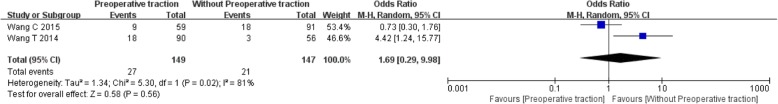
Meta-analysis results of the correlation between preoperative traction and ONFH after IFFNF.

Information in 2 [16, 19] of the references over preoperative traction was included in meta-analysis by the random effect model due to a significant statistical heterogeneity (*I*^2^ = 81%, *P* = 0.02). The results shown that there was nonsignificant differences in preoperative traction between ONFH patients and normal ones (OR:1.69, 95%CI: 0.29–9.98, *P* = 0.56). (Fig. 7).

#### Status of implants

**Fig. 8:**
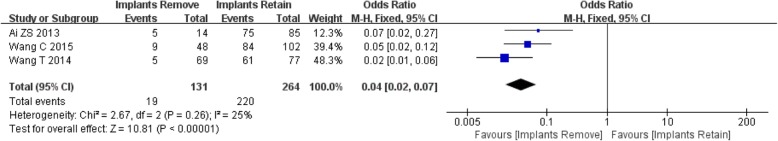
Meta-analysis results of the correlation between status of implants and ONFH after IFFNF.

Three studies [16, 19, 23] reporting statistics on the status of implants were involved in meta-analysis. There was no significant statistical heterogeneity among the studies (*I*^2^ = 25%, *P* = 0.26) and the fixed effect model was utilized. It was found that the difference in implants status was significant between the two patient groups (OR: 0.04, 95%CI: 0.02–0.07, *P* < 0.00001). (Fig. 8).

#### Mechanism of injury

**Fig. 9:**
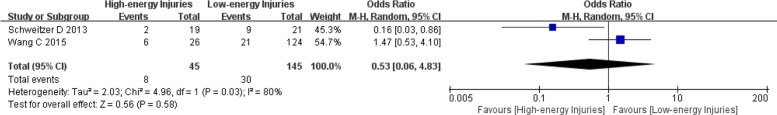
Meta-analysis results of the correlation between mechanism of injury and ONFH after IFFNF.

Two references [19, 21] explored the underlying mechanism of the injury. The relevant data were analyzed by the random effect model, for a significant statistical heterogeneity was found among the studies (*I*^2^ = 80%, *P* = 0.03). There was a significant difference in the mechanism between ONFH patients and normal ones (OR: 0.53, 95%CI: 0.06–4.83, *P* = 0.58). (Fig. 9).

#### Sensitivity analyses

**Fig. 10:**
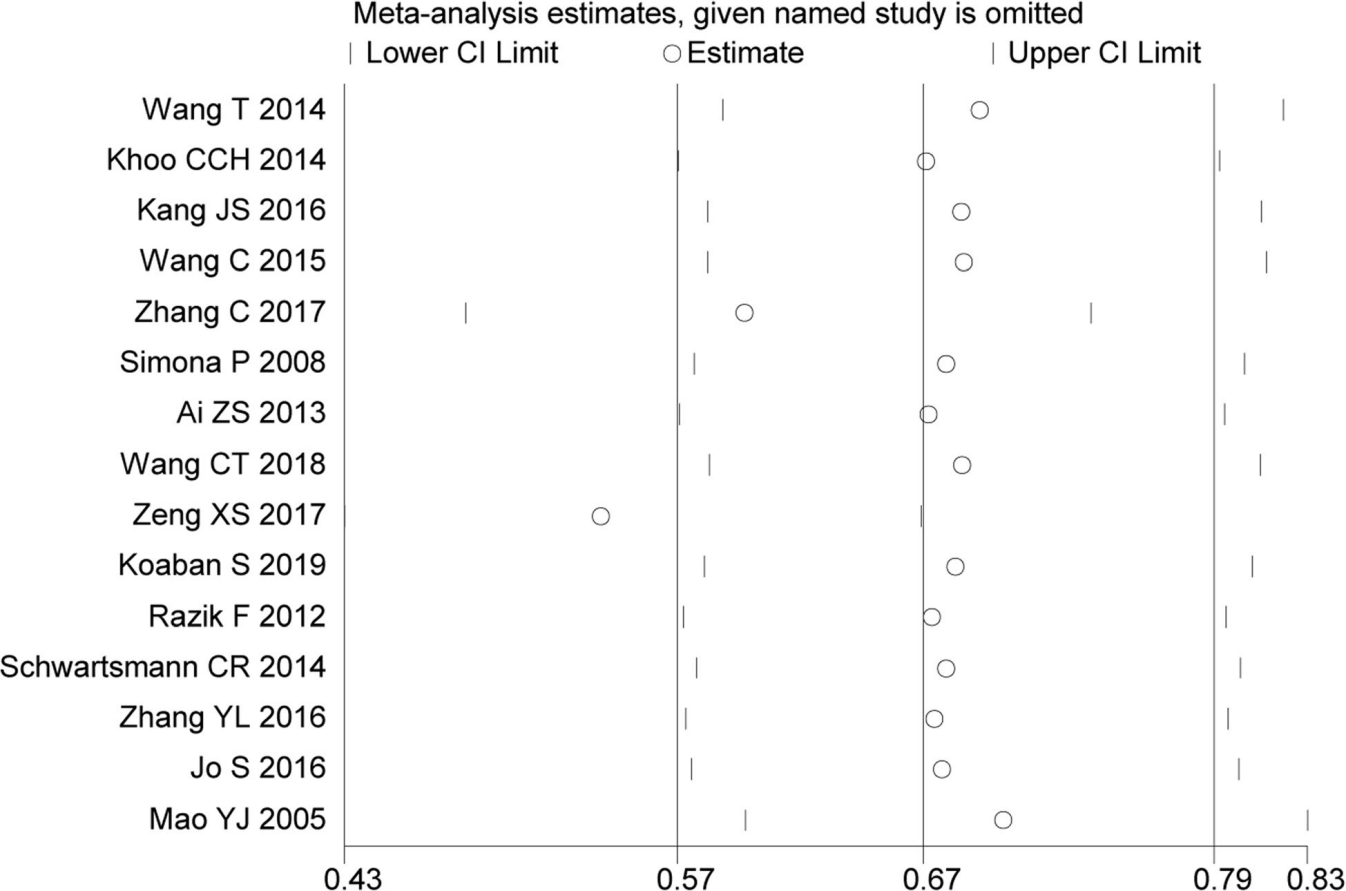
Influence analysis of included studies

The sensitivity analysis was performed on the selected studies to assess whether individual studies would affect the overall results. The outcomes suggested that data of one study [25] strongly affected the overall results (Fig. 10). Rereading the article verbatim, we found that the study actually accepted 150 cases with ONFH of 325 in total; and the incidence was 46.15%, much larger than the others, averaged 22.95%. Therefore, we discarded this reference from the meta-analysis.

#### Evaluation of publication bias

**Fig. 11:**
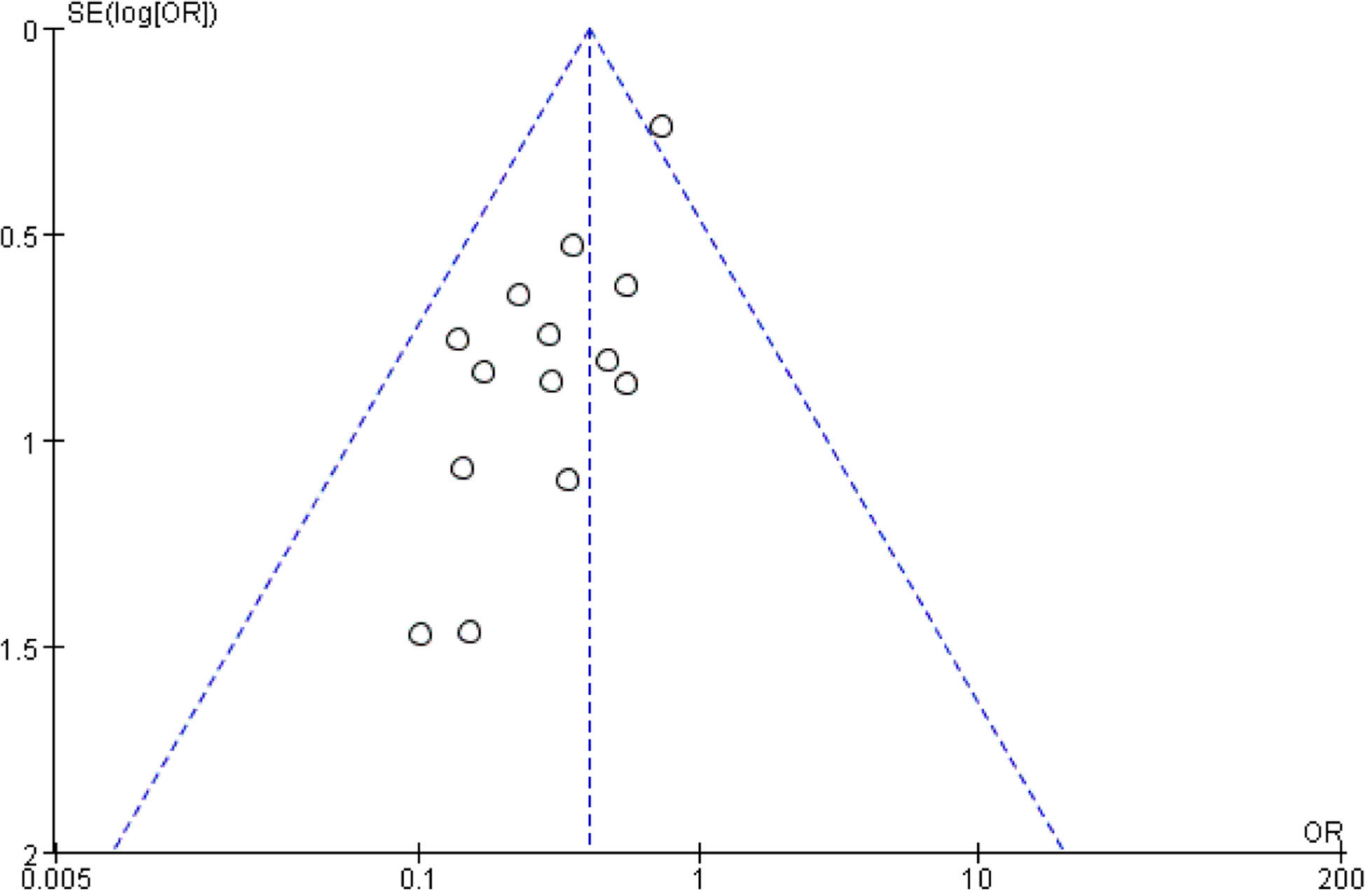
Funnel plot of correlation between Garden classification and ONFH after IFFNF.

**Fig. 12:**
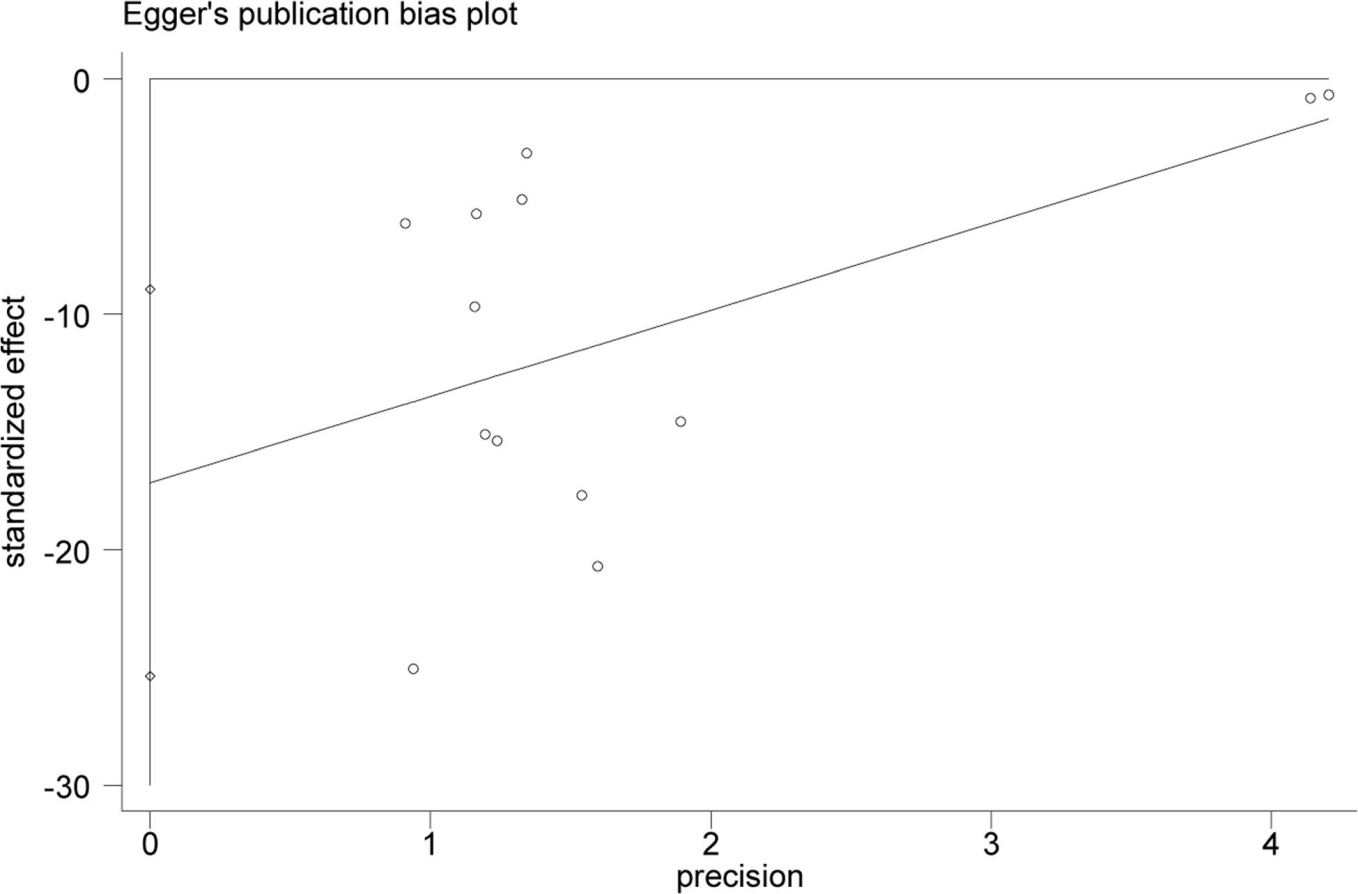
Egger’s funnel plot

**Fig. 13:**
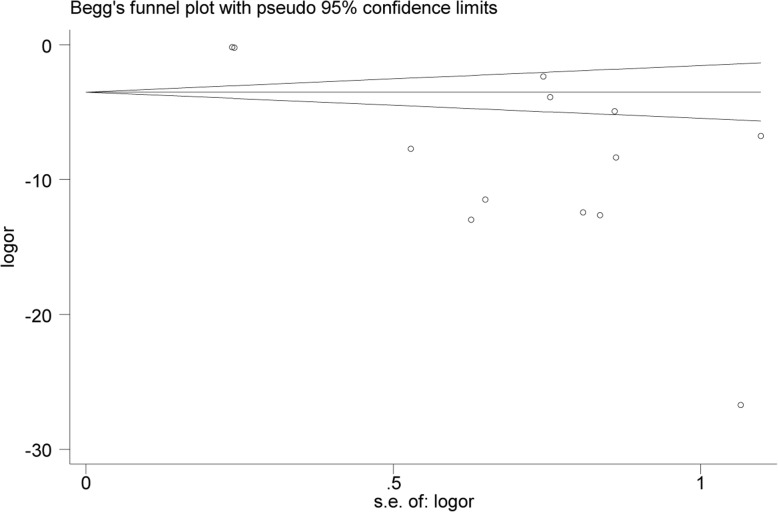
Begg’s funnel plot

Visual inspection of funnel plots was adopted in the estimation. Specifically, Egger and Begg tests uncovered publication bias in this study (*P* = 0.01). This may attribute to incomplete retrieval or unpublished negative results. (Figs. 11, 12 and 13).

## Discussion

In this study, we conducted a meta-analysis of 16 selected studies to corroborate the risk factors of ONFH after IFFNF. To ensure a reliable conclusion, previous published studies on ONFH after IFFNF were retrieved, reviewed and summarized to achieve those with high compliance and high quality, so as to answer various clinical questions of this malady. Overall, our results suggest that Garden analysis and the retention of internal fixators are critical risk factors of ONFH after IFFNF. Gender, age, injury-operation interval, fracture reduction method, preoperative traction and mechanism of ONFH cannot be considered as correlative factors of ONFH after IFFNF. In addition, the publication bias was found by the visual distribution of funnel plots and Egger and Begg tests, indicating incomplete retrieval or unpublished negative results. The sensitivity analysis indicated one research, by Zeng XS et al [25], had significant influence on the overall results, so the study was excluded.

Orthopaedic surgeons undoubtedly encounter great challenges on the treatments for FNF, for contentions between distinct therapies have persisted for years. Generally, the commonly acceptable treatment is IF by open or closed reduction for young patients or ones without degenerative changes in the hip. Total hip arthroplasty is usually the preferable option for patients with extant hip degenerative changes. The primary purpose of IF for the fractures under this condition is to achieve anatomic reduction, which is salutary to restore or maintain the unstable blood supply to the femoral head and prevent complications (e.g., ischemic necrosis and non-healing) in the days to come.

Garden classification is considered as an important factor in the formation of ONFH after IFFNF. Strong relationship is reported between the risks of avascular necrosis in displaced intracapsular fractures and undisplaced FNF [33–35]. Sun et al [36] suggested that screw removal might be a primary inducing factor in the pathogenesis of ONFH, because all compressive, tensile and shear stresses were concentrated on the fracture site after screw removal, changing the biological stress thereby. Yao S et al [37] suggested that the intraosseous pressure of the femoral head could increase in that the presence of the screw exacerbated the femoral head ischemia.

As the passages have exposed, two significant advantages of our study are clear. Firstly, as the risk factors of ONFH after IFFNF were controversial, this meta-analysis assessed the potential correlation between ONFH and ten more indexes (gender, age, Garden classification, etc.) after IFFNF through a thorough systematic study with rigorous analytical methods. Secondly, the rationality and reliability of our meta-analysis have been prudently and significantly improved in that the overall comprehensive estimation is based on a large sample size. In addition, sufficient sensitivity analysis has been carried out to ensure the reliability of this study.

The current meta-analysis has the following limitations and must be considered before our results are accepted. First, the selected studies in the meta-analysis were published between 2005 and 2019. The influencing factors reported in the studies were not completely consistent, and most of them did not adjust for confounding factors. Second, the values of meta-analysis in this study were derived from univariate analysis, with residual confounding and unmeasured factors. Our more accurate results will come from adjustments for other confounding factors. Third, the research included in this analysis is insufficient, and potential publication bias still exists. Fourth, this study only includes references in English. Therefore, we may have lost data from those in other languages.

## Conclusion

In summary, our meta-analysis suggests that Garden classification and the retention of IF are important factors affecting ONFH after IFFNF. However, because of the publication bias of this study and the results from the univariate analysis, we cannot determine whether the two items are independent risk factors of ONFH after IFFNF. In this study, gender, age, injury-operation interval, fracture reduction mode, preoperative traction and the mechanism of ONFH have been ruled out from the consideration to be correlative factors with this complication after IFFNF.

## Data Availability

All data generated or analysed during this study are included in this published article.

## Abbreviations

FNF: Femoral neck fracture
IF: Internal fixation
MeSH: Medical Subject Headings
NOS: Newcastle-Ottawa Scale
ONFH: Osteonecrosis of femoral head
PRISMA: Preferred Reporting Items for Systematic Reviews and Meta-Analyses
